# Single Chain Antibody Fragment against Venom from the Snake *Daboia russelii formosensis*

**DOI:** 10.3390/toxins9110347

**Published:** 2017-10-27

**Authors:** Chi-Hsin Lee, Yu-Ching Lee, Yueh-Lun Lee, Sy-Jye Leu, Liang-Tzung Lin, Chi-Ching Chen, Jen-Ron Chiang, Pharaoh Fellow Mwale, Bor-Yu Tsai, Ching-Sheng Hung, Yi-Yuan Yang

**Affiliations:** 1 Graduate Institute of Medical Sciences, College of Medicine, Taipei Medical University, Taipei 11031, Taiwan; chihsine@msn.com (C.-H.L.); yllee@tmu.edu.tw (Y.-L.L.); cmbsycl@tmu.edu.tw (S.-J.L.); ltlin@tmu.edu.tw (L.-T.L.); 2 The Center of Translational Medicine, Taipei Medical University, Taipei 11031, Taiwan; ycl@tmu.edu.tw; 3 Department of Microbiology and Immunology, School of Medicine, College of Medicine, Taipei Medical University, Taipei 11031, Taiwan; 4 Department of Pathology and Laboratory Medicine, Landseed Hospital, Taoyuan 32449, Taiwan; chen3971@landseed.com.tw; 5 Center for Diagnostics and Vaccine Development, Centers for Disease Control, Ministry of Health and Welfare, Taipei 11561, Taiwan; jrc@cdc.gov.tw; 6 School of Medical Laboratory Science and Biotechnology, College of Medical Science and Technology, Taipei Medical University, Taipei 11031, Taiwan; pmwale@medcol.mw; 7 Navi Bio-Therapeutics Inc., Taipei 10351, Taiwan; boryutsai@navibio.com.tw; 8 Department of Laboratory Medicine, Wan Fang Hospital, Taipei Medical University, Taipei 11696, Taiwan; oryx@w.tmu.edu.tw; 9 Core Laboratory of Antibody Generation and Research, Taipei Medical University, Taipei 11031, Taiwan

**Keywords:** *Daboia russelii formosensis* (DRF), IgY antibody, phage display technology, single-chain variable fragment (scFv) antibody

## Abstract

Russell’s vipers containing hemotoxic and neurotoxic venom commonly cause snake envenomation. Horse-derived antivenom is a specific antidote, but its production is expensive and has side effects. Developing a cost-effective and more tolerable therapeutic strategy is favorable. In this study, using glutaraldehyde-attenuated *Daboia russelii formosensis* (DRF) venom proteins to immunize chickens, polyclonal yolk-immunoglobulin (IgY) antibodies were generated and showed a specific binding affinity. Phage display technology was used to generate two antibody libraries of single-chain variable fragments (scFvs) containing 3.4 × 10^7^ and 5.5 × 10^7^ transformants, respectively. Phage-based ELISA indicated that specific clones were enriched after bio-panning. The nucleotide sequences of scFv-expressing clones were analyzed and classified into six groups in the short linker and four groups in the long linker. These scFv antibodies specifically bound to DRF proteins, but not other venom proteins. Mass spectrometric data suggested that these scFv antibodies may recognize phospholipase A2 RV-4 or RV-7. In vivo studies showed that anti-DRF IgY exhibited complete protective effects and mixed scFv antibodies increased the survival rate and time of mice challenged with a lethal dose of DRF proteins. These antibodies can be potentially applied in a rapid diagnostic method or for treatment in the future.

## 1. Introduction

Snake envenomation is considered a major medical problem worldwide, particularly in tropical or subtropical countries, such as Taiwan. Globally, venomous snake bites cause approximately 125,000 deaths each year [1,2]. Because bites can occur in secluded areas or are self-treated without medical attention, many cases are most likely unreported. Russell’s viper (*Daboia russelii*), a common venomous snake widely distributed in South and Eastern Asia, can be classified into five subspecies based on their characteristics and colorations. These subspecies include *Daboia russelii formosensis* in Taiwan; *Daboia russelii siamensis* in Thailand, Myanmar and China; *Daboia russelii russelii* (previously named *Daboia russelii nordicus*) in North India; *Daboia russelii pulchella* in Sri Lanka and South India; and *Daboia russelli limitis* in Indonesia and Java [3,4,5]. Due to the significant variations in the components of snake venom proteins associated with geographic regions, victims often present various clinical symptoms caused by bites of different subspecies of Russell’s viper [3,6]. In Taiwan, *D. r. formosensis*, also known as the chain snake, is one of the most common causes of snake envenomation. *D. r. formosensis* venom (DRF proteins) contains a complex of proteins with different biological functions, such as phospholipase A2 (PLA2) [7], activated factor V and hemorrhagins and neurotoxins, which cause hemolysis, renal failure and neurotoxicity [8,9]. In the presence of all of the components, PLA2 with different isoenzymes is considered one of the major lethal components in crude DRF venom proteins and affects cardiotoxicity, myotoxicity and antiplatelet activity [10,11]. Therefore, development of therapeutic agents against specific components is limited. Thus far, horse antivenom has been the most common antidote available for treating snake envenomation. However, antivenom production in horses requires a high cost that includes rearing horses and refining IgG antibodies from serum. In addition, horse antivenom occasionally causes side effects, such as serum sickness or anaphylactic shock [12]. Therefore, alternative therapeutic strategies, including cost-effective antivenom production and rapid diagnostic methods, against snake envenomation are necessary to act as adjuvants and prophylaxes to existing anti-snake venom treatments.

To solve the problems associated with antibody production in horses, chickens might be an alternative to mammals as antibody producers because they are inexpensive to raise and easy to handle [13]. The production of large amounts of polyclonal immunoglobulin from the yolk of chicken eggs (so-called IgY antibodies) is easy and does not require bleeding to purify antibodies [14]. Each egg contains 100–150 mg of IgY antibodies, and approximately 2–10% of the total yield of IgY antibodies is antigen-specific [15]. In addition, the problems encountered during the collection and preparation of snake venom proteins could be settled because only a small amount of antigens is required to elicit a strong humoral immune response in chickens, thus making them an ideal alternative for producing antigen-specific antibodies [16]. Otherwise, studies have reported that using IgY antibodies with neutralizing activity and without negative side effects as a passive immunization might be a cheaper alternative therapeutic strategy [17,18]. Thus, it is encouraging that chickens are economic hosts for producing neutralizing antibodies against snake envenomation.

However, because polyclonal antibodies, including IgY, contain a panel of antibodies with diverse activities, their specificity to targeted antigen is often low, leading to reduced efficacy of antibody treatment or application for diagnostic reagents. Furthermore, cross-reactions occasionally cause harmful side effects when polyclonal antibodies are applied. Thus, the quality and quantity of polyclonal antibodies vary profoundly depending on the production methods and are also limited by the size and lifespan of animals [19]. By contrast, monoclonal antibodies secreted by a single B cell clone specifically recognize only one epitope, thus making them highly specific and less cross-reactive. Monoclonal antibodies have been widely used in basic research, clinical diagnosis, and therapeutics [20,21]. Although the efficacy of one monoclonal antibody against one epitope might be lower than polyclonal antibodies against many epitopes when used in neutralizing snake venom proteins, a combination of various monoclonal antibodies still has the potential to neutralize snake venom proteins to reduce symptoms, increase survival time, and even prevent death [22]. Specific monoclonal antibodies can also be used as a rapid diagnostic test on wound exudates from victims to ascertain the type of snake bite [23]. Presently, hybridoma and phage display antibody technologies are the two major systems used to generate monoclonal antibodies. The traditional hybridoma technology requires a cumbersome and expensive process [24]. Alternatively, phage display technology is convenient, rapid and inexpensive when used in selecting specific antibodies from constructed antibody libraries [25]. A small amount of antibody fragments, such as single chain variable fragment (scFv) or antigen-binding fragment (Fab), is especially suitable for generation using phage display technology. A monoclonal scFv antibody, which is composed of a light chain variable domain (V_L_) and heavy chain variable domain (V_H_) linked by a flexible peptide linker, contains monovalent affinity of the parent IgG [26]. Currently, small antibody fragments are emerging as new tools for diagnosis and therapy in the biotechnology market. 

The aforementioned studies showed the need for a more convenient and cost-effective host to replace traditional generation of neutralizing antibodies in horses. In addition, monoclonal antibodies have a strong specificity that can more precisely determine the type of venomous snake bite allowing for the choice of appropriate treatments. Chickens are appropriate, convenient and widely available hosts for generating polyclonal and monoclonal antibodies in many areas [27,28]. In this study, high titers of polyclonal IgY antibodies were first elicited in DRF protein-immunized chickens and monoclonal scFv antibodies were later generated using phage display technology. Both IgY and scFv antibodies were characterized using Western blot and enzyme-linked immunosorbent assay (ELISA). The results showed that most scFv antibodies specifically recognized DRF proteins but did not cross-react with other snake venom proteins. Moreover, when injected into mice challenged with a lethal dose of DRF proteins, IgY antibodies prevented the death of mice while scFv antibodies also reduced mortality and prolonged the survival time in mice. Therefore, we believe that specific polyclonal IgY and monoclonal scFv antibodies have great potential to be applied in the development of diagnostic agents and therapeutics for venomous snake bites after further modifications.

## 2. Results

### 2.1. Characterization of Anti-DRF IgY against DRF Proteins

**Figure 1:**
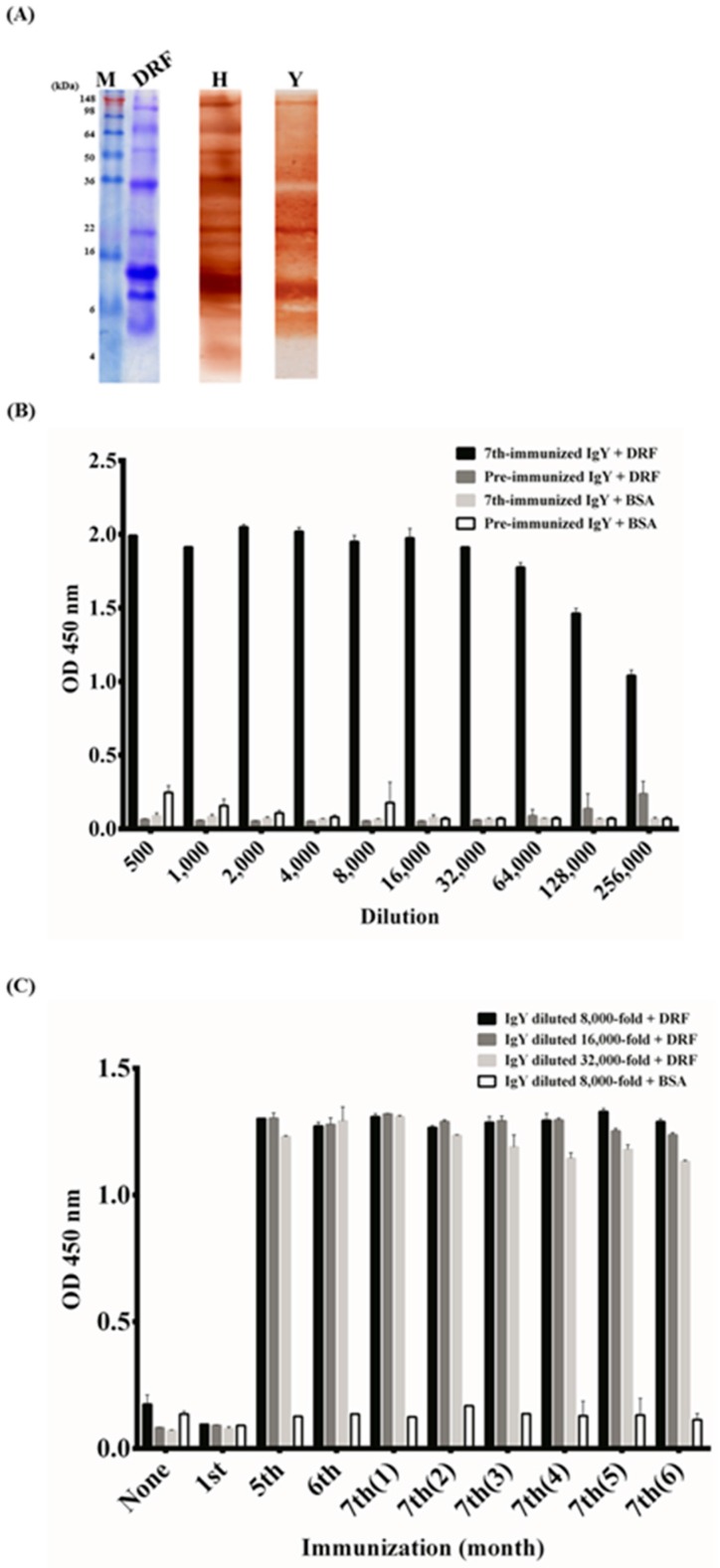
Characterization of the polyclonal anti-*Daboia russelii formosensis* (DRF) IgY antibodies: (**A**) DRF proteins were analyzed on Coomassie brilliant blue-stained SDS-PAGE (lane DRF). After transfer onto blots, DRF proteins were detected by horse-derived antivenom (lane H) or polyclonal IgY antibodies from chickens after the 7th immunization (lane Y). (**B**) Purified IgY from pre-immunized chickens or from chickens immunized seven times in a series of two-fold dilution (500×–256,000×) were tested for their binding specificity to DRF and BSA proteins on ELISA wells, respectively. (**C**) Humoral antibody response in chickens was monitored for six months after the 7th immunization. Lane M contained protein markers.

DRF venom proteins were separated and analyzed on Coomassie BRILLIANT BLUE stained SDS-PAGE (Figure 1A, lane DRF). Three major proteins with molecular weights of approximately 35 kDa, 13 kDa and 8 kDa were visualized. Horse-derived antivenom (lane H) and purified IgY (lane Y) antibodies from chickens immunized seven times showed similar binding patterns, with the most significant visualization of a 13 kDa protein on Western blots. The ELISA results also indicated that 256,000-fold diluted polyclonal IgY antibodies bound strongly to DRF proteins (ODs > 0.8), but not to BSA (Figure 1B). To monitor the humoral response in chickens, IgY antibodies were purified from eggs collected from chickens at various stages of immunization for ELISA. The results indicated that a significant antibody response was elicited after the 5th immunization, reached a plateau and was maintained for at least six months thereafter (Figure 1C). 

### 2.2. Construction of Phage Libraries Displaying scFv Antibodies

Total RNA was extracted from sacrificed chicken spleens to synthesize cDNA copies. To obtain full-length scFv genes, two consecutive rounds of PCR were performed. In the first PCR, the V_H_ and the V_L_ fragments were amplified to be approximately 400 bps and 350 bps, respectively (data not shown). The amplified V_L_ and V_H_ products were joined and extended by overlapping PCR to form full-length scFv fragments containing a short or long linker (scFv-S or scFv-L) (data not shown), which were later cloned into phagemid and transformed into *E. coli*. The sizes of the two antibody libraries were estimated to contain 3.4 × 10^7^ and 5.5 × 10^7^ transformants, respectively. After infection by the M13 helper phage, the recombinant phages displaying scFv antibodies were used for bio-panning later. 

### 2.3. Bio-Panning for Selecting Specific scFv Antibodies

**Figure 2:**
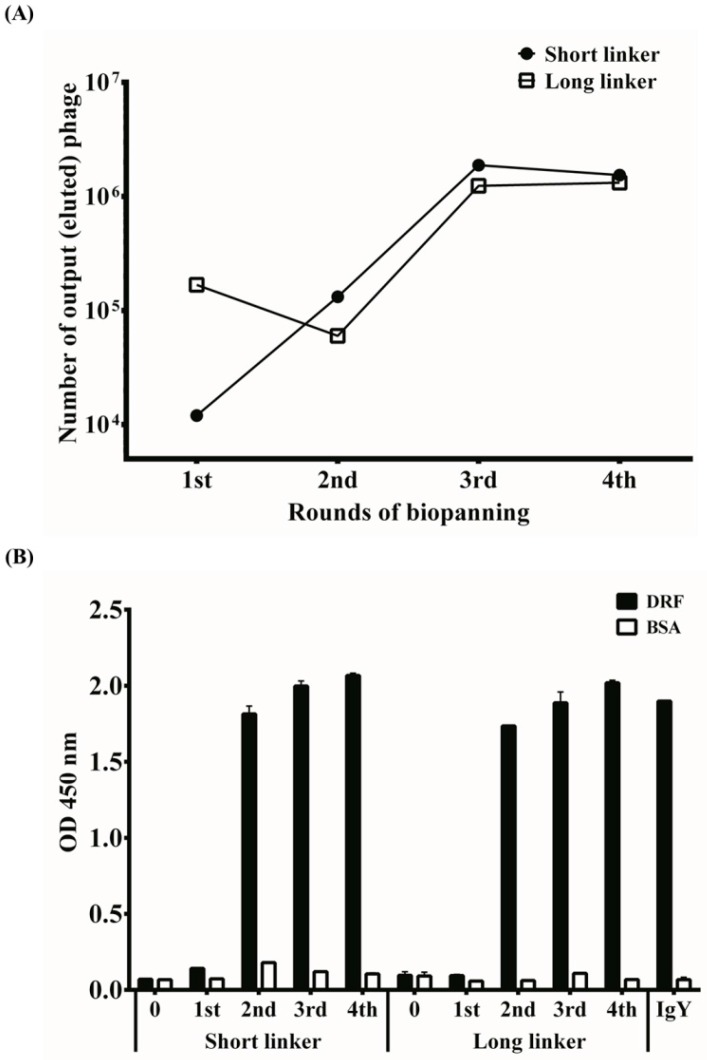
Determination of the phage titers and their specific binding activity. (**A**) eluted phage titers were determined after each round of bio-panning using a plaque formation assay and monitored for four rounds; and (**B**) amplified phages (10^11^–10^12^ pfu) from each round of bio-panning were examined for their binding against DRF and BSA proteins on ELISA wells. Partially purified anti-DRF IgY was used as a positive control.

Four cycles of bio-panning were performed to select specific monoclonal scFv antibodies against DRF venom proteins. After each round of bio-panning, the titers of eluted phages were determined (Figure 2A). The eluted phage titer of the scFv-S library was approximately 10^4^ colony-forming units (CFU) in the first bio-panning and increased steadily thereafter. By contrast, the eluted phage titer of the scFv-L library was approximately 10^5^ CFU in the first bio-panning and decreased to 7 × 10^4^ CFU in the second bio-panning. We do not know the exact reasons for this observation. For both libraries, the titers increased in the third bio-panning and reached a plateau of approximately 10^6^ CFU. These results were normally encountered as demonstrated by our previous studies, indicating that phages with specific anti-DRF activities were enriched throughout the bio-panning steps. This result was further verified using phage-based ELISA. The amplified phages after each round of bio-panning were examined for their binding activities to DRF venom proteins on ELISA plates. IgY antibodies with anti-DRF activities were included as a positive control. As seen in Figure 2B, the results showed that the binding activities of recombinant phages significantly increased in the second round of bio-panning and remained at a plateau stage. No reactivity to BSA was detected. 

### 2.4. Sequence Analysis, Expression and Purification of scFv Antibodies

**Figure 3:**
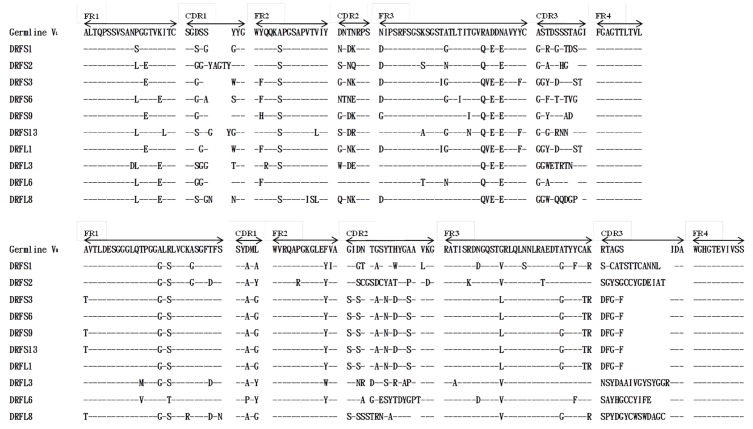
Sequence alignment of the *V_H_* and *V_L_* domains of selected monoclonal anti-DRF scFv antibodies. Nucleotide sequences of 10 representative clones (six scFv-S with a short linker and four scFv-L with a long linker) after the 4th bio-panning were determined. The putative amino acid sequences were aligned with those of a chicken germline gene. Sequence gaps were introduced to maximize the alignment by blank spaces. The dashes (–) indicate identical amino acid sequences. The boundaries of framework regions (FRs) and complementarity determining regions (CDRs) are indicated above germline amino acid sequences.

First, a total of 40 clones from scFv-S and scFv-L libraries (20 clones/each) were randomly selected to express scFv antibodies, and all of them expressed scFv antibodies (data not shown). Based on the expression patterns on Western blots, 15 clones from each library were further subjected to nucleotide sequence determination of the *V_L_* and *V_H_* genes. Alignment with the chicken immunoglobulin germline gene showed that six distinct groups of scFv antibodies with a short linker were identified and represented by DRFS1 (7/15; 46.67%), DRFS2 (2/15; 13.33%), DRFS3 (3/15; 20%), DRFS6 (1/15; 6.67%), DRFS9 (1/15; 6.67%) and DRFS13 (1/15; 6.67%). Similarly, four groups of scFv antibodies with a long linker were identified and represented by DRFL1 (6/15; 40%), DRFL3 (3/15; 20%), DRFL6 (3/15; 20%) and DRFL8 (3/15; 20%) (Table 1). The deduced amino acid sequences of the *V_L_* and *V_H_* regions of these scFv antibodies were aligned with those of the chicken germline (Figure 3). Compared with the framework regions (FRs), the variability in the complementarity-determining regions (CDRs) were much greater, particularly that of CDR3s, which had 22–80% and 50–100% mutation rates in the *V_L_* and *V_H_* genes, respectively (Table 2). In the *V_L_* domains, CDR1 and CDR2 showed the same lengths with those of chicken germline, except that DRFS2 had five additional amino acids, DRFS3 and DRFL1 had one less amino acid, DRFS13 had two additional amino acids, and DRFL8 had one additional amino acid in CDR1. In the V_H_ domains, the sequences in CDR2s were more variable (31–61%) than those in CDR1s (40%). More importantly, CDR3s in both the *V_L_* and *V_H_* regions showed the most variability (22–80% and 50–100%), including the sequences and lengths, compared to those of the chicken germline. DRFS3, DRFS9 and DRFS13 with an identical *V_H_* gene were referred to as cluster A, whereas the DRFS6 and DRFL1 with an identical *V_H_* gene were referred to as cluster B. Interestingly, only one difference in the first amino acid (T to A) in FR1 of the *V_H_* genes used by these scFv antibodies in clusters A and B was observed. The biological significance(s) of a threonine residue in place of alanine is presently not known. 

**Figure 4:**
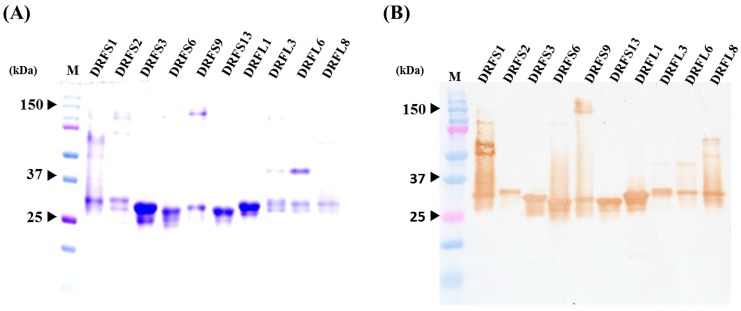
Purification and analysis of 10 representative anti-DRF scFv antibodies: (**A**) after IPTG induction, scFv antibodies (lanes DRFS1 to DRFL8) containing a His-tag peptide were purified by Ni^2+^ Sepharose and visualized on SDS-PAGE; and (**B**) the identity of these scFv antibodies was further verified by probing with goat anti-chicken light chain antibody on Western blots.

After inducing with IPTG overnight, these 10 recombinant His-tagged scFv antibodies were purified using Ni^2+^ Sepharose and analyzed via SDS-PAGE and Western blots. A major band with slight variation in molecular weight (approximately 30 kDa) was visualized, demonstrating that these ten scFv antibodies were expressed properly and highly purified (Figure 4A). The expressed scFv antibodies were also recognized by anti-chicken light chain antibodies (Figure 4B). However, in addition to the major band, scFv antibodies prepared from DRFS9 and DRFL8 clones also contained proteins with larger molecular weights both on SDS-PAGE and Western blots. The identity of these proteins was not known, but they were considered to be the aggregated form of scFv antibodies. Their identity could potentially be determined after elution with His-elution buffer containing 6 M urea during antibody purification (data not shown). 

### 2.5. Specific Binding Assay of Selected scFv Antibodies

**Figure 5:**
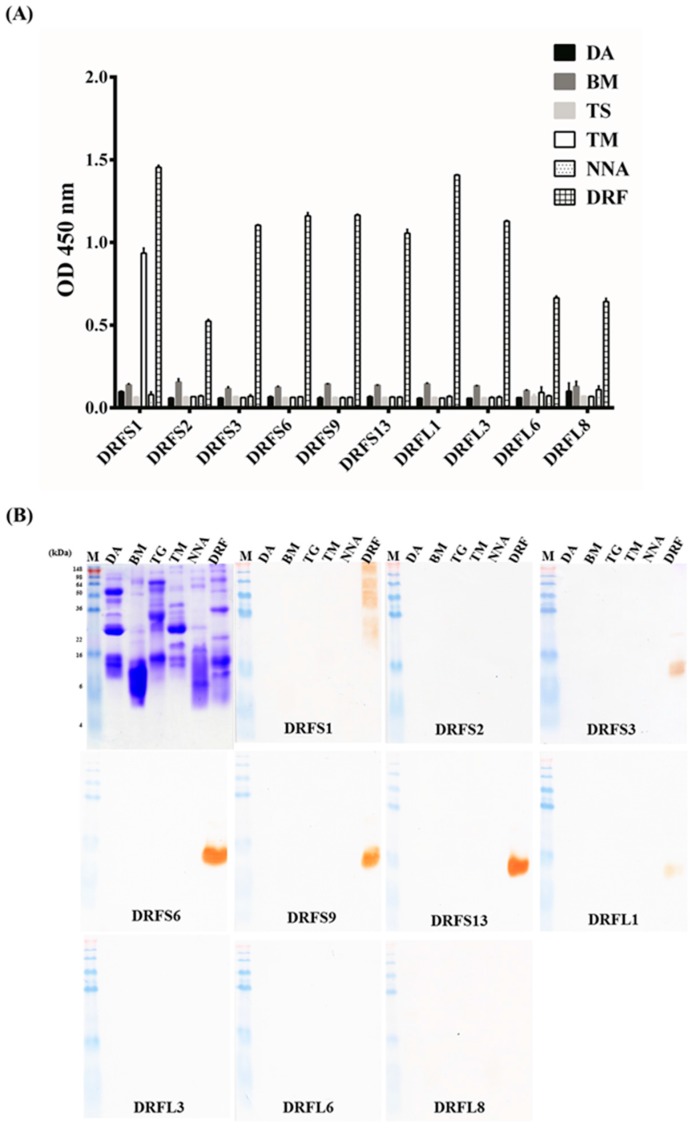
Binding assay of 10 representative anti-DRF scFv antibodies to various snake venom proteins: (**A**) venom proteins from six species of snakes (DA, BM, TS, TM, NNA and DRF) were immobilized on ELISA plates and detected by purified scFv antibodies (5 μg/mL); and (**B**) binding specificity of these antibodies was further examined as shown on the Western blots.

Venom proteins from six major venomous snakes (DA, BM, TS, TM, NNA and DRF) were immobilized on ELISA plates or PVDF membranes and detected by the purified scFv antibodies. The ELISA results showed that the 10 purified scFv antibodies exhibited various levels of binding activities to DRF proteins (Figure 5A). DRFS2, DRFL6 and DRFL8 scFv had weak binding activities (ODs 0.5 to 1.0), while DRFS1, DRFS3, DRFS6, DRFS9, DRFS13, DRFL1 and DRFL3 had strong binding activities (ODs > 1.0). Interestingly, DRFS1 also showed significant cross-reactivity with TM venom proteins (Figure 5A). Moreover, the binding specificities of these 10 scFv antibodies were further confirmed on Western blots (Figure 5B). DRFS3, DRFS6, DRFS9, DRFS13 and DRFL1 scFv antibodies recognized a protein with a molecular weight of 14 kDa at various degrees, while DRFS2, DRFS3, DRFL6 and DRFL8 showed no detectable signals. However, it was unexpected to observe that DRFS1 reacted with several venom proteins (Figure 5B). In addition, it was an interesting observation that DRFL3 had a stronger binding activity on ELISA but no signals on Western blots. It is possible that DRFL3 recognized antigenic epitopes on DRF proteins that were denatured on Western blots. Taken together, these results indicate that the DRFS1, DRFS3, DRFS6, DRFS9, DRFS13, DRFL1 and DRFL3 scFv antibodies recognized DRF proteins with high specificity. 

### 2.6. Competitive Inhibition Assay by ELISA

**Figure 6:**
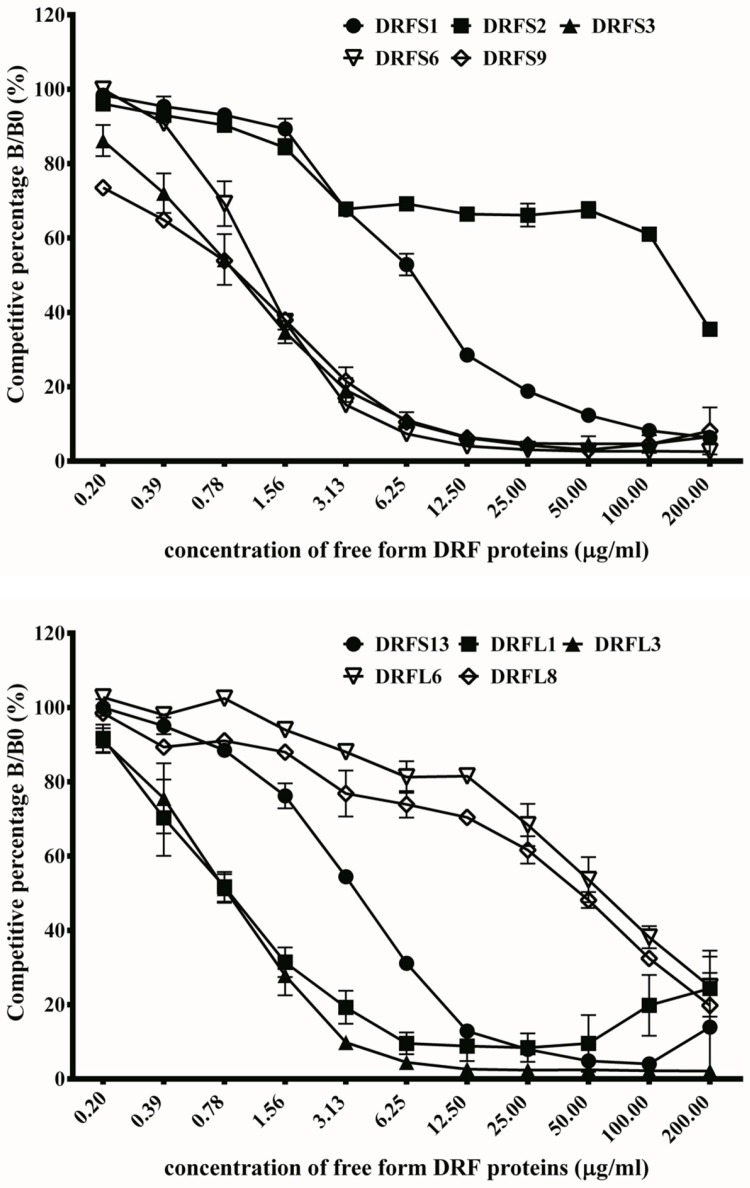
Inhibition of anti-DRF scFv antibodies against DRF proteins. Before addition to ELISA plates immobilized with DRF proteins, purified scFv antibodies were pre-incubated with different concentrations of soluble DRF proteins as described in the text. The competitive percentage was shown as B/B0, representing the amounts of bound scFv in the presence or absence of DRF proteins, respectively. ELISA data were shown as the means of duplicated experiments.

To further confirm the binding activities of these scFv antibodies, competitive ELISAs were performed. The 10 scFv antibodies were individually mixed with free forms of DRF venom proteins, which were later added to ELISA wells coated with DRF proteins. The absorbance values in the absence of free forms of DRF proteins were used at 100% to calculate the percentage of inhibitory effects against those in the presence of different concentrations of free forms of DRF proteins. The binding activities of these scFv antibodies to DRF proteins were inhibited in a dose-dependent manner (Figure 6). The calculation showed that only 1.56 μg/mL of the free forms of DRF proteins was required to achieve more than 60% inhibition on anti-DRF activities of DRFS3 (65%), DRFS6 (63%), DRFS9 (62%), DRFL1 (69%) and DRFL3 (72%) scFv antibodies. Otherwise, 12.5, 200, 6.25, 100 and 100 μg/mL of the free forms of DRF proteins were required to achieve inhibitory effects on anti-DRF activities of DRFS1 (71%), DRFS2 (65%), DRFS13 (69%), DRFL6 (62%) and DRFL8 (67%). Moreover, the amount of DRF proteins required to reach 50% of the inhibitory effects for the binding activities of DRFS3, DRFS6, DRFS9, DRFS13, and DRFL1 were 0.52, 0.63, 0.51, 2.04, and 0.48 μg/mL, respectively. Thus, the dissociation constant (K_d_) values of these four scFv antibodies were calculated to be 3.7 × 10^−8^, 4.5 × 10^−8^, 3.6 × 10^−8^, 1.5 × 10^−8^ and 3.4 × 10^−8^ M based on the Klotz plot method [29]. Taken together, these results demonstrate that DRFS3, DRFS6, DRFS9, DRFL1 and DRFL3 possessed the strongest and yet similar anti-DRF binding activities, while DRFS2 was the weakest binder. 

### 2.7. Mass Spectrometry Analysis of Bound DRF Proteins

**Figure 7:**
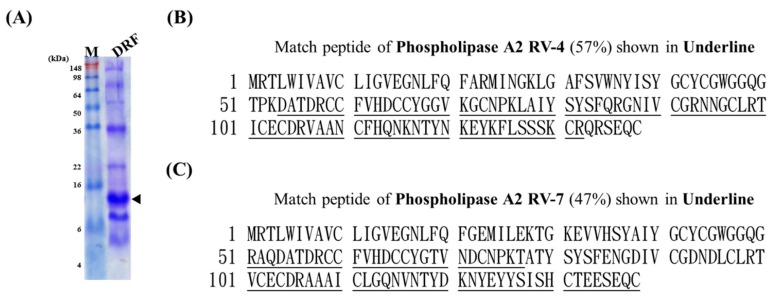
Mass spectrometric analysis of DRF proteins recognized by anti-DRF scFv antibodies. (**A**) The major protein (marked by an arrow) recognized by DRFS3, DRFS6, DRFS9, DRFS13 and DRFL1 on Western blot was located, excised from the SDS-PAGE gel, and subjected to mass spectrometry. Amino acid sequence alignment showed that the identified protein contained 57% and 47% homology with PLA2: RV-4 (**B**); and RV-7 (**C**) present in DRF proteins, respectively, as shown by the underlined peptides.

To characterize the protein recognized by DRFS3, DRFS6, DRFS9, DRFS13 and DRFL1, a band with approximately 14 kDa was identified, excised and subjected to mass spectrometric analysis (as arrowed in Figure 7A). The alignment data showed that these trypsin-digested fragments could be mapped to amino acid residues 54–132 of phospholipase A2 (PLA2) RV-4 with a 57% homology in amino acid sequences (Figure 7B). The identified protein also showed a 47% homology to PLA2 RV-7, spanning amino acids 51–77 and 101–138 (Figure 7C). These results indicated that the DRFS3, DRFS6, DRFS9, DRFS13 and DRFL1 scFv antibodies might recognize the antigenic epitopes on PLA2 enzymes, which are a large family in DRF venom proteins.

### 2.8. In Vivo Neutralization Assay

**Figure 8:**
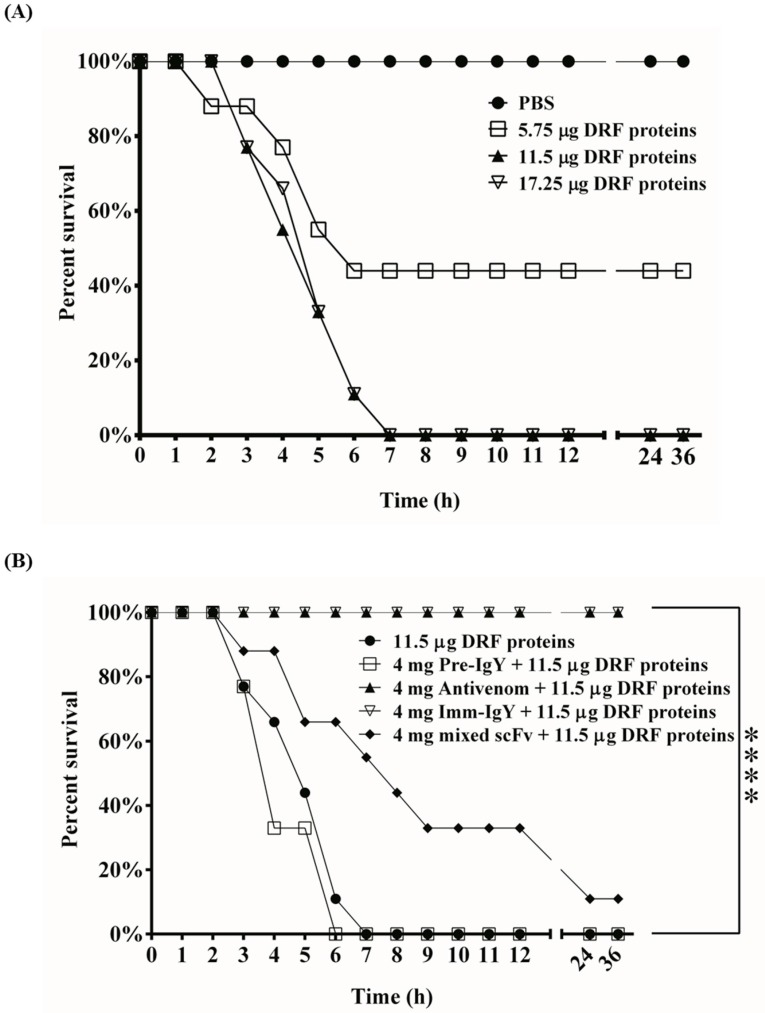
Neutralization assay of anti-DRF scFv antibodies in vivo: (**A**) groups of nine ICR mice were intraperitoneally administered with different doses of DRF proteins (5.75, 11.5 and 17.25 μg) to determine the MLD; and (**B**) mice were challenged with polyclonal IgY antibodies from pre-immunized (Pre-IgY) or immunized (Imm-IgY) chickens, horse-derived antivenom (Antivenom), or a mixture of 10 anti-DRF scFv antibodies (4 mg), all of which were individually pre-mixed with DRF proteins at 37 °C for 1 h. The treated mice were monitored at 1 h intervals for 36 h. The survival rates were analyzed via Gehan-Breslow-Wilcoxon test. **** *p* < 0.0001.

To ascertain the MLD, we injected mice with 5.75, 11.5 or 17.25 μg of DRF venom proteins intraperitoneally (Figure 8A). The results showed that administration of 5.75 μg of DRF proteins led to the death of two mice within 2 to 4 h, two within 5 h and one within 6 h. Four survived without any notable abnormality. By contrast, all mice injected with either 11.5 μg or 17.25 μg of DRF proteins died within 7 h, while complete survival rates were observed in PBS-administered mice (Figure 8A). Consequently, 11.5 μg of DRF proteins was considered as 1× MLD and used for neutralization studies. As shown in Figure 8B, 4 mg of IgY antibodies from pre-immunized chickens provided no protection, leading to 100% death of mice treated with 1× MLD of DRF proteins within 6–7 h. By contrast, 4 mg of anti-DRF IgY from 7th-immunized chickens or horse-derived antivenom provided full protection in mice with the identical treatment. We further analyzed the neutralizing effect of 4 mg of a mixture of ten anti-DRF scFv antibodies on mice. The results showed that four mice died within 7 h, two survived for 1–2 h longer, two for more than 5 h longer, and one survived throughout the experiments, suggesting that the mixture containing 10 anti-DRF scFv antibodies could partially neutralize the lethal effect and significantly prolong the survival time on mice administered with DRF venom proteins. 

## 3. Discussion

To generate neutralization antibodies, collecting snake venom proteins is a major limitation, including collecting DRF venom proteins. According to the World Health Organization (WHO) public protocol [2,30], horses are immunized with snake venom proteins at 1–4 mg/horse for the first immunization and at 5–10 mg/horse for subsequent immunizations with a two-week interval until the enzyme immunoassay titers plateau. In this study, the initial immunization was 100 μg/chicken and subsequent immunizations were 80 μg/chicken at one-week intervals. We performed seven immunizations with a total of approximately 600 μg of venom proteins for two months. Our method is more cost-effective than production of horse antivenom, which requires approximately 60 mg for two months. Previous studies also indicated that it is 30–40% less expensive to use chickens as an immunoglobulin source instead of horses [31]. Based on our results, a strong antibody response was elicited in chickens and lasted for at least six months according to Western blot (Figure 1A) and ELISA assays (Figure 1B,C). The immunization doses were adequate to generate neutralizing antibodies according to the in vivo neutralization assay (Figure 8B), confirming a small amount of venom proteins was sufficient to generate neutralizing IgY antibodies in chickens, which are difficult to collect and refine for the preparation antivenom antibodies in various seasons and regions.

Since being developed by Smith [32], phage display technology has proven to be a useful platform for studying protein–protein interactions in immunology, pharmacology, drug discovery, and antibody generation [33]. Chickens offer an inexpensive and widely available alternative to generate monoclonal antibodies with the support of a phage display system and could be a potentially valuable source of antibodies for therapies [25,34]. Chickens are suitable for use in the phage display system because of their relatively unsophisticated immunoglobulin repertoire because it is easier to generate antibody libraries from a single association of V_L_ and V_H_ recombination [35]. In this study, we constructed two scFv antibody libraries containing 3.4 × 10^7^ and 5.5 × 10^7^ transformants from chickens immunized with DRF venom proteins. The construction of these recombinant phage antibody libraries was time-saving and effectively yielded specific anti-DRF scFv antibodies as observed on phage-based ELISA plates after two bio-panning rounds (Figure 2). These results further demonstrate that the production of monoclonal scFv antibodies with high specificity using phage display antibody libraries from hyperimmunized animals is faster and more efficient than most naïve libraries, which require 4–6 or more bio-panning rounds to achieve [36]. The size of the library required to produce highly specific antibodies using hyperimmune animals as the source of immunoglobulin cDNA is reduced rather than using naïve libraries [37].

As mentioned above, sequence analysis of putative amino acids of anti-DRF scFv antibodies randomly selected after the fourth bio-panning clearly revealed significant variations in V_L_ and V_H_ gene usage in these clones, which were classified into six short linker groups and four long linker groups, as summarized in Figure 3 and Table 1. Only one amino acid difference (T to A) was identified in the FR1 domain of the V_H_ genes used by scFv antibodies in clusters A (DRFS3, DRFS9, DRFS13) and B (DRFS6, and DRFSL1). In addition, DRFS3, DRFS6, DRFS9, DRFS13 and DRFSL1 scFv antibodies possessing different V_L_ genes were not only expressed at various levels under same experimental condition (Figure 4A) but also exhibited distinct binding activities against DRF venom proteins on ELISA and Western blots (Figure 5A,B). For example, the DRFSL1 scFv antibody showed a significant binding signal on ELISA but was much weaker on Western blots. Collectively, we speculated that the V_L_ genes, but not V_H_ genes, may play major roles in the expression levels of scFv antibodies and in their binding activities and the differential recognition of antigenic epitopes against DRF proteins. This speculation is contrary to previous reports that indicate that the V_H_ genes of chicken IgY antibodies are critical for antigen-binding specificity [38]. However, it needs more investigation to demonstrate our speculation.

It is well documented that the length of the CDR3 domains in the V_H_ genes of chicken IgY is extremely broad, from 8 to 32 amino acids (mean 16.2 ± 3.2), and are similar to those of humans (5–37 amino acids, mean 16.1 ± 4.1), 89% of which contain 15–23 amino acids [38,39]. In comparison, the scFv antibodies in our study had 8–18 amino acids in the CDR3 of V_H_, 70% of which contained less than 15 amino acids, as evident in those of DRFS3, DRFS6, DRFS9, DRFS13 and DRFL1 (8 amino acids) and DRFS2 and DRFL6 (14 amino acids). However, our results did not provide any clue as to whether the lengths of the CDR3 domains in the V_H_ genes of anti-DRF scFv antibodies significantly contribute to their functional activities. Additionally, previous reports indicated that functional V_H_ or V_L_ genes generated through V-D-J or V-J recombination and somatic mutation were mutually assorted to create more antibody diversity in most vertebrates [40,41]. Sequence comparison of the V_H_ or V_L_ genes of anti-DRF scFv antibodies with those of the germline gene revealed high mutation rates in the amino acids of all six CDR domains. As shown in Figure 3 and Table 2, the mutation rates in the CDR domains of the V_H_ or V_L_ genes ranged 40–73% and 17–58%, respectively. By contrast, the mutation rates in the FR domains of the V_H_ or V_L_ genes ranged 7–13% and 9–14%. More importantly, the mutation rates found in the CDR3 of the V_H_ genes of all ten scFv antibodies were 50% to 100%. These results were consistent with those of previous reports, supporting the fact that somatic hypermutations occurred to increase affinity more often in the CDR domains than in the FR of the rearranged functional antibodies [41,42]. Thus, our results also suggest that these anti-DRF scFv antibodies were generated and selected as a result of affinity maturation of B cells through an antigen-driven response in the chickens immunized with venom proteins. However, it can be disputed that the promiscuous pairing of V_L_ and V_H_ genes often occurred in *E. coli* cells, arguing that the anti-DRF scFv antibodies were not generated from authentic antigen-stimulated B cells in chickens [25]. Such a problem could be circumvented only by performing further experiments in the future. 

The DRFS2, DRFL3, DRFL6 and DRFL8 scFv antibodies did not show detectable signals against DRF proteins on Western blots even though they showed moderate to strong binding activities on ELISA. These results suggest that they recognized conformational structures rather than linear epitopes present on the venom proteins. In addition to anti-DRF binding activity, DRFS1 scFv also bound to TM venom proteins on ELISA (Figure 5A). By contrast, it only reacted with DRF proteins in smear patterns but not with TM proteins on Western blots (Figure 5B). The underlying mechanism is not presently understood. It was assumed that DRFS1 scFv may recognize antigenic epitope(s) conserved in several DRF venom proteins or in one protein with different post-translational modifications. Moreover, the cross reactivity against both TM and DRF venom proteins suggested that DRFS1 scFv may recognize snake venom serine proteases or phospholipase A2 proteins, which share a high degree of homology in amino acid sequences, as suggested previously [43,44,45]. However, this speculation could be only confirmed with further characterization. The DRFS3, DRFS6, DRFS9, DRFS13 and DRFL1 scFv antibodies also showed a significant binding signal on ELISA (Figure 5A). Interestingly, they all recognized a protein with a molecular weight of 14 kDa on Western blots, although the recognition signal shown by DRFL1 was barely detected (Figure 5B). Again, this observation may result from differential presentation of epitope(s) using the two methods. Though its identity needs further characterization, the protein was estimated to be 25% of the crude DRF venom proteins as analyzed by ImageJ software [46]. 

The bound protein was subjected to mass spectrometric analysis and showed 57% and 47% homology with the amino acid sequence of PLA2 RV-4 or RV-7, respectively (Figure 7). The PLA2 enzymes containing a large number of homologous proteins with approximately 13–15 kDa are the major components in the venom of the Viperidae family, including Russell’s viper [47,48,49]. Russell’s viper venom had several PLA2 isoenzymes, which could be classified as acidic, basic, and neutral, with similar structures. The enzymes induced various pathophysiological effects, such as neurotoxicity, cardiotoxicity, hemorrhage, and edema, in bitten victims [49,50]. A previous study indicated that heterodimeric viperotoxin F formed by PLA2 RV-4 and RV-7, accounting for approximately 40% of the crude venom, were considered the major lethal constituents of the venom from *D. r. formosensis* [7,51]. Although RV-4 and RV-7 had approximately 65% identity in amino acid sequences [51], RV-4 is known to be neurotoxic, whereas RV-7 is not [7]. However, RV-7 is considered to facilitate the specific binding of RV-4 to the presynaptic binding sites. In such a context, knowing that a mixture of our scFv antibodies prolonged the survival of DRF-treated mice (Figure 8B), we concluded that some of these scFv antibodies may reduce the neurotoxic effect of DRF venom by directly blocking the active domains of RV-4 or RV-7 or inhibiting the formation of viperotoxin F protein. However, additional studies are required to further confirm the exact underlying mechanism of the inhibitory effects. Because the polyclonal anti-DRF IgY antibodies showed complete protection in mice (Figure 8B), it is believed that additional anti-DRF scFv antibodies in the constructed libraries with neutralizing activities against the DRF proteins could be identified. Accordingly, as mentioned above, all of the anti-DRF scFv antibodies together would have great potential for the development of therapeutic treatments against snake envenomation in the future.

## 4. Materials and Methods

### 4.1. Animal Models 

All animal experiments were approved by the Institutional Animal Care and Use Committee of the Taipei Medical University before project initiation. Female White Leghorn (*Gallus domesticus*) chickens at 6 months of age and ICR mice weighing 12–14 g were purchased from the National Laboratory Animal Center, Taiwan. All animals were maintained in the animal core facility of the Taipei Medical University. (Ethical approval code: LAC-99-0133; valid on 1 January 2011).

### 4.2. Chicken Immunization

DRF protein powders, kindly provided by the Centers for Disease Control (CDC) in Taiwan, were dissolved in phosphate-buffered saline (PBS). Before immunization, DRF proteins were mixed with 0.125% glutaraldehyde (GA; Sigma-Aldrich, Saint Louis, MO, USA) in the dark for 1 h at room temperature (25 °C) [52]. For the first immunization, 100 μg of DRF proteins was mixed with an equal volume of complete Freund’s adjuvant and administered into different areas of chicken thighs intramuscularly. For subsequent immunizations, 80 μg of DRF proteins mixed with an equal volume of incomplete Freund’s adjuvant were administered at weekly intervals. Eggs were collected before immunization, each immunization, and each month after the 7th immunization to purify polyclonal IgY antibodies using dextran sulfate and sodium sulfate as described previously [53,54]. 

### 4.3. Construction of Antibody Libraries

Construction of antibody libraries was performed as previously described [55]. Briefly, chickens after seven rounds of immunization were sacrificed to extract total RNA from the spleens, which were homogenized in 5 mL of Trizol solution (Invitrogen, Carlsbad, CA, USA) according to the manufacturer’s protocol. The cDNA was synthesized using 20 μg of total RNA by reverse transcriptase, and used to amplify V_L_ and V_H_ genes of IgY immunoglobulins using chicken-specific primers. The amplified V_L_ and V_H_ genes were linked by a 7-(GQSSRSS) or an 18-(GQSSRSSSGGGSPGGGGS) amino acid peptide linker to form functional scFv antibody genes by overlapping extension PCR. After SfiI (New England Biolabs, Ipswich, CA, USA) restriction, the scFv fragments were ligated into the pComb3X vector, which were later transformed into the Escherichia coli (*E. coli*) ER2738 strain by electroporation (MicroPulser, Bio-Rad, Hercules, CA, USA). A small portion of the transformed *E. coli* cells was plated on LB agar plates containing 50 μg/mL of ampicillin (Amp) to estimate the size of the constructed antibody library. The remaining *E. coli* cells were added to 100 mL of super broth and infected by 10^12^ plaque forming units (pfu) of VCS-M13 helper phage overnight. After centrifugation, the recombinant phages in the supernatant were precipitated by adding 4% polyethylene glycol (PEG) 8000 and 3% NaCl on ice. After another centrifugation, the recombinant phages were re-suspended in 1× PBS containing 1% bovine serum albumin (BSA) and 20% glycerol. The titers of the recovered phages were determined and stored at −20 °C until use. 

### 4.4. Bio-Panning for Selecting scFv Antibodies

Four rounds of bio-panning were performed on 96-well microplates. Briefly, DRF venom proteins (0.5 μg) were immobilized on wells at 4 °C overnight and blocked with 3% BSA at 37 °C for 1 h, followed by adding recombinant phages (10^11^–10^12^ pfu) and incubating at 37 °C for 2 h. Unbound phages were washed out using PBST (1× PBS containing 0.05% Tween 20), and bound phages were eluted using 0.1 M glycine–HCl (pH 2.2) by vigorous pipetting. After neutralization using 2 M Tris base buffer, the eluted phages were used to infect ER2738 E. coli cells for amplification. A portion of the transfected cells was plated on LB agar plates containing 50 μg/mL of Amp to determine the titers of eluted phages. The remaining infected cells were cultured overnight and the amplified phages were collected for the next round of bio-panning. 

### 4.5. Protein Expression and Purification of scFv Antibodies

Total phagemid DNA from the 4th bio-panning was purified and transformed into TOP10F’ *E. coli* to analyze individual scFv antibodies. Overnight cultures of randomly selected clones were diluted 100× in super broth medium containing 20 mM MgCl_2_ and 50 μg/mL of Amp and further incubated at 37 °C for 8 h, followed by the addition of 1 mM isopropyl-β-d-thiogalactopyranoside (IPTG) for overnight induction. Bacteria were collected by centrifugation, re-suspended in histidine (His) binding buffer (20 mM sodium phosphate, 0.5 M NaCl, 20 mM imidazole, pH 7.4), and lysed by sonication. Ni^2+^ Sepharose (GE Healthcare BioSciences AB, Uppsala, Sweden) was used to purify His-tagged scFv antibodies according to the manufacturer’s instructions. The purified scFv antibodies were dialyzed against 1× PBS and concentrated using Amicon Ultra-4 Centrifugal Filter Units (Merck Millipore, Darmstadt, Germany). 

### 4.6. Western Blotting 

DRF proteins on SDS-PAGE were transferred to polyvinylidene fluoride (PVDF) membranes, which were blocked with 5% skim milk in 1× PBS at 25 °C for 1 h. The membranes were incubated with commercially available horse antivenom at 1:1000 dilution at 25 °C for 1 h and washed three times with PBST, followed by the addition of horseradish peroxidase (HRP)-conjugated goat anti-horse Fab (Jackson ImmunoResearch, West Grove, PA, USA). After three washings, bound horse anti-DRF antibodies on the membranes were visualized by adding diaminobenzidine (DAB). Similarly, the anti-DRF binding activity of purified polyclonal IgY antibodies from chickens from pre- and post-immunization were examined and detected using HRP-conjugated donkey anti-chicken IgY (Jackson ImmunoResearch, West Grove, PA, USA). Additionally, purified scFv antibodies (5 μg/mL) were incubated with membranes containing venom proteins of *Deinagkistrodon acutus* (DA), *Bungarus multicinctus* (BM), *Trimeresurus stejnegeri* (TS), *Trimeresurus mucrosquamatus* (TM), *Naja naja atra* (NNA), and DRF proteins. To detect the bound anti-DRF scFv antibodies, goat anti-chicken light chain IgG (Bethyl, Laboratories, Montgomery, TX, USA) and HRP-conjugated donkey anti-goat IgG (Jackson ImmunoResearch, West Grove, PA, USA) were used. Other steps, such as blocking, washing, incubation, and color development steps, were performed as described above.

### 4.7. Enzyme-Linked Immunosorbent Assay (ELISA) and Competitive ELISA

DRF and BSA proteins (0.5 μg/well) were immobilized on ELISA plates at 37 °C for 1 h, which were blocked with 1× PBS containing 5% skim milk for an additional hour. Then, purified IgY from pre-immunized chickens or from chickens immunized 7 times were 2× serially diluted (500×–256,000×) and added to incubate for 1 h. After washing three times with PBST, HRP-conjugated donkey anti-chicken IgY was added and incubated at 37 °C for 1 h. After three additional washings as above, the binding activities were detected by adding 3,3′,5,5′-tetramethylbenzidine (TMB). The reactions were stopped using 1 N HCl, and the optical readings were taken at 450 nm. In phage-based ELISA, 10^11^–10^12^ pfu of amplified phages from each round of bio-panning was added to the wells immobilized with DRF proteins, and detected by HRP-conjugated mouse anti-M13 antibodies (GE Healthcare Bio-Sciences, Marlborough, MA, USA). To further verify the specific binding activities, the selected scFv antibodies (5 μg/mL) were added to the ELISA wells immobilized with venom proteins of DA, BM, TS, TM, NNA, and DRF. Subsequently, goat anti-chicken light chain IgG and HRP-conjugated donkey anti-goat IgG antibodies were used for detection. 

For competitive ELISA, DRF proteins in a series of 2× dilutions (400 μg/mL to 0.40 μg/mL) were mixed with an equal volume of scFv antibodies (10 μg/mL). After incubation at 25 °C for 1 h, the mixtures were added to the DRF protein-coated wells and incubated at 37 °C for another 1 h. The blocking, washing, incubation, and color development were performed following the same conditions as described above. All of the ELISA data were represented as the mean ± SD from two independent experiments.

### 4.8. Sequence Analysis of Selected scFv Antibodies

The nucleotide sequences of *V_L_* and *V_H_* of scFv antibody genes from randomly selected clones was determined using primer ompseq (5′-AAGACAGCTATCGCGATTGCAGTG-3′) by the ABI 3730 XL autosequencer machine (Applied Biosystems, Foster City, CA, USA). The deduced amino acid sequences of scFv antibodies representing frameworks (FRs) and complementarity determining regions (CDRs) were aligned and analyzed with that of the chicken immunoglobulin germline gene using the BioEdit alignment program [33].

### 4.9. Mass Spectrometric Analysis

DRF proteins were separated on SDS-PAGE and visualized by Coomassie brilliant blue staining. A major protein commonly recognized by DRFS3, DRFS6, DRFS9, DRFS13 and DRFL1 scFv antibodies was identified, excised from the gel and subjected to mass spectrometric analysis by LTQ Orbitrap XL MS (Thermo FisherScientific, Waltham, MA, USA) after trypsin digestion. The SwissProt 2011 database (533,049 sequences; 189,064,225 residues) was used for computational analysis.

### 4.10. Neutralization Assay of Antibodies against DRF Proteins

A neutralization assay was performed according to the WHO protocol [2,34]. ICR mice were randomly allocated to several groups of nine. To determine the minimum lethal dose (MLD), preparations of 5.75, 11.5, and 17.25 μg of DRF venom proteins were individually dissolved in 200 μL of 1× PBS, incubated at 37 °C for 1 h, and then intraperitoneally injected into the mice. PBS containing no DRF proteins was included as a control. For the antibody neutralization assay, IgY from pre-immunized chickens or from chickens immunized 7 times (4 mg/each), horse-derived antivenom (4 mg) or a mixture of ten anti-DRF scFv antibodies (4 mg) were incubated with 11.5 μg of DRF proteins in 200 μL of PBS at 37 °C for 1 h, respectively. Subsequently, the resulting mixtures were intraperitoneally injected into the mice. The status of the mice was monitored hourly for 36 h.

### 4.11. Statistical Analyses 

The neutralization assays in mice were analyzed via the Gehan–Breslow–Wilcoxon test using GraphPad Prism 6 software (La Jolla, CA, USA). A *p* value of < 0.05 was considered statistically significant.

## Figures and Tables

**Table 1:** Classification of anti-DRF scFv clones according to the identity of *V_L_* and *V_H_* regions.

Groups	Short Linker			Long Linker		
	*V_L_*	*V_H_*	Percentage	*V_L_*	*V_H_*	Percentage
Group 1	1, 4, 7, 8, 12, 14, 15	1, 4, 7, 8, 12, 14, 15	46.67%	1, 2, 4, 5, 11, 15	1, 2, 4, 5, 11, 15	40%
Group 2	2, 5	2, 5	13.33%	3, 9, 10	3, 9, 10	20%
Group 3	3, 10, 11	3, 9, 10, 11, 13	20%	6, 7, 12	6, 7, 12	20%
Group 4	6	6	6.67%	8, 13, 14	8, 13, 14	20%
Group 5	9		6.67%			
Group 6	13		6.67%			

**Table 2:** Amino acid mutation rates of single-chain variable fragment (scFv) clones.

Region	CDR1	CDR2	CDR3	Total CDRs	FR1	FR2	FR3	FR4	Total FRs
*V_L_*	13~54%	0~57%	22~80%	17~58%	5~15%	6~25%	9~25%	0%	9~14%
*V_H_*	40%	31~61%	50~100%	40~73%	7~20%	0~14%	6~19%	0%	7~13%

CDRs, complementarity determining regions; FRs, framework regions; *V_L_*, variable region in light chain; *V_H_*, variable region in heavy chain.
